# The prevalence of Q fever in the Eastern Mediterranean region: a systematic review and meta-analysis

**DOI:** 10.4178/epih.e2022097

**Published:** 2022-10-28

**Authors:** Mozhgan Ahmadinezhad, Leila Mounesan, Amin Doosti-Irani, Manijeh Yousefi Behzadi

**Affiliations:** 1 Department of Epidemiology and Biostatistics, School of Public Health, Tehran University of Medical Sciences, Tehran, Iran; 2 Department of Epidemiology and Biostatics, Research Centre for Emerging and Reemerging Infectious Diseases, Pasteur Institute of Iran, Tehran, Iran; 3 Department of Epidemiology, School of Public Health and Research Center for Health Sciences, Hamadan University of Medical Sciences, Hamadan, Iran; 4 National Reference Laboratory of Plague, Tularemia and Q Fever, Research Centre for Emerging and Reemerging Infectious Diseases, Pasteur Institute of Iran, Akanlu, KabudarAhang, Hamadan, Iran

**Keywords:** Mediterranean region, Q fever, Meta-analysis, Prevalence, *Coxiella burnetii*

## Abstract

**OBJECTIVES:**

Q fever, caused by the bacterium, is a major zoonotic disease around the world. This disease is common in the Eastern Mediterranean region; therefore, we conducted the first systematic review and meta-analysis on its prevalence in humans, animals, and ticks in the Eastern Mediterranean region.

**METHODS:**

Major Iranian and international databases were searched from 2000 to 2021. We extracted the prevalence of Q fever in blood samples from animals and milk samples from animals, ticks, and humans as the main outcome. We reported the prevalence of seropositivity and molecular positivity as point estimates and 95% confidence intervals (CIs).

**RESULTS:**

In this review, 112 papers were identified. The overall seroprevalence of Q fever was 22.4% (95% CI, 19.8 to 25.1). The pooled prevalence of Q fever in ticks was 17.5% (95% CI, -1.3 to 36.4). The prevalence was 25.5% (95% CI, 16.1 to 34.9) in humans. The prevalence of Q fever in animal blood samples from goats, sheep, camels, cattle, cats, dogs, horses, and buffalo were 28.1%, 25.1%, 25.0%, 20.1%, 9.8%, 8.4%, 6.5%, and 6.3%, respectively. Furthermore, the prevalence of Q fever in milk samples of animals was higher in cattle (20.3%) than in sheep (20.0%), goats (16.4%), and camels (3.3%).

**CONCLUSIONS:**

*Coxiella burnetii* infections are common in humans and in a wide range of animal species, but they are still not recognized in many countries in the Eastern Mediterranean region, thus presenting a significant threat to human and animal health in the region.

## INTRODUCTION

Emerging and re-emerging infectious diseases are recognized worldwide as a threat to public health. More than half of these are zoonotic diseases [1] that are often transmitted to humans through direct contact with animals, their carcasses, and animal-derived products [2].

Q fever is a zoonotic disease [3] that leads to public health problems worldwide and is especially common in developing countries. This disease is caused by *Coxiella burnetii* and infects humans, wild animals, and domestic animals [4]. The *C. burnetii* bacterium grows in the environment and enters the body of ticks and mites [5,6]. The infection then enters the body of domestic ruminants (mainly cattle, sheep, and goats), which constitute the most important reservoir of *C. burnetii* [7,8].

Q fever is mostly asymptomatic in animals, except in cases where it causes miscarriage, stillbirth, and infertility [9]. Humans are also susceptible to this disease. The main route of transmission of Q fever to humans is through inhalation of contaminated aerosols or dust containing *C. burnetii* [10]. Additionally, human contact with feces, urine, embryos, and placentas of infected animals can transmit the disease [11,12]. However, humans can also contract *C. burnetii* through biting and consuming dairy products [13]. In humans, the clinical manifestations of *C. burnetii* infection include acute or chronic syndromes [14]. The most common form of the disease is similar to the flu. The clinical manifestations include fever, headache, coughing, atypical pneumonia, hepatitis, myalgia, arthralgia, cardiac involvement, skin rash, and neurological signs, and 2% of patients with the acute form of the disease are hospitalized [15]. This disease has imposed substantial costs on patients and health systems [16].

Many countries in the world are exposed to Q fever. However, in some regions, the prevalence of this disease is different; for instance, in low-income countries such as the Eastern Mediterranean region (EMR), this disease has become a health problem [17- 19]. Review studies conducted in Iran [20], Pakistan [21], and Tunisia [22] have studied the prevalence of Q fever in animals, humans, and dairy products. However, there has not yet been a systematic review and meta-analysis that compiles and reviews all relevant studies in this field in the EMR.

Despite variation in certain health and disease indices, countries of the EMR region have similar cultural, economic, and medical characteristics. Furthermore, addressing the prevalence of Q fever would be useful for designing effective intervention strategies to control the disease. Therefore, the purpose of this systematic review and meta-analysis was to conduct a comprehensive epidemiological study of Q fever in the EMR.

## MATERIALS AND METHODS

### Literature search strategy

Initially, we searched PubMed, ISI Web of Science, and Scopus databases as major international databases, and Magiran and SID for Persian-language articles. These national databases cover Iranian scientific journals.

The keywords that we used for our search were “Q fever,” “*Coxiella burnetii*,” “query fever,” and “Pakistan,” “Afghanistan,” “Bahrain,” “Djibouti,” “Egypt,” “Iran,” “Iraq,” “Jordan,” “Kuwait,” “Lebanon,” ”Libya,” “Morocco,” “Oman,” “Qatar,” “Somalia,” “Saudi Arabia,” “Syria,” “Sudan,” “Tunisia,” “United Arab Emirates” and “Yemen” in English sources. For the Iranian databases (Magiran and SID), we used both English and Persian keywords.

We searched the databases for articles that reported the prevalence of Q fever in humans, ticks, and mites, and in milk and blood samples from animals such as cattle, sheep, goats, camels, buffalo, and horses, cats, and dogs in the EMR. We started the search in the year 2000 because an increased interest has been seen in Q fever research in the EMR since this year. All articles reporting *C. burnetii* prevalence in humans or animals by any serological or molecular method were included in the study. The researchers used enzyme-linked immunosorbent assays and indirect immunofluorescence assays to diagnose Q fever through serological methods and polymerase chain reaction as a molecular method.

### Eligibility criteria and study selection

We first performed the screening using titles first then abstracts and full texts of the studies were reviewed independently by 2 authors (MA and MYB). Any disagreement between these 2 authors was resolved by a third author (ADI). The authors of this meta-analysis reviewed the studies based on established inclusion criteria.

The inclusion criteria included studies measuring the prevalence of Q fever in ticks and mites, humans, and animal specimens (including cattle, sheep, goats, camels, buffalo, dogs, cats, and horses). Studies conducted in the countries of the EMR region from 2000 to 2021 were included in our meta-analysis.

The exclusion criteria included (1) letters, books, editorials, reports, and reviews; (2) studies where the place of sample collection and its origin were not known; (3) studies that did not clearly state the sample size and positive cases; and (4) studies in countries other than the EMR.

### Data extraction

We used a pre-designed template to extract data from the imported articles. The extracted data included disease/pathogen, year, country, design, species/occupation, number of animals/humans/samples tested, method of diagnosis, study outcomes, and the first author of each study. Data extraction was conducted independently by the same 2 review authors (MA and MYB) who conducted the study selection.

### Quality assessment

The quality of papers was assessed using the Newcastle-Ottawa quality assessment scale (NOS) designed for human observational studies [23]. The NOS consists of 3 domains: the selection of study groups, comparability of groups, and description of exposure and outcome.

This quality assessment tool includes 6-8 items and star scores for each study in each domain. All items have 1 star except the comparability domain (the maximum score based on stars for the comparability domain is 2). The total score of each article was calculated. Then, all the selected studies were categorized as high (5-7), medium (4-3), or low quality (< 3). Two authors (MA and ADI) reviewed the articles separately. The opinion of the third author was used to address any issues of disagreement.

### Statistical analysis

We estimated the prevalence of Q fever with 95% confidence intervals (CIs) by subgroups of species and country. Statistical heterogeneity was explored using the I^2^ statistic. We adopted a random-effects model to estimate the prevalence of Q fever and performed subgroup analyses by the species of data collection, country, year of publication, and occupation. To explore the main factors influencing prevalence estimates and sources of heterogeneity, we conducted a meta-regression analysis for species, country variables, year of publication, and occupation. Publication bias was assessed using the Egger and Begg tests, with p-value < 0.05 indicating significant bias. The analysis was performed using Stata version 16 (Stata Corp., College Station, TX, USA).

### Ethics statement

Ethical approval was not sought because this study was based on published articles and no human or animal intervention was performed.

## RESULTS

### Study characteristics

**Figure 1.**
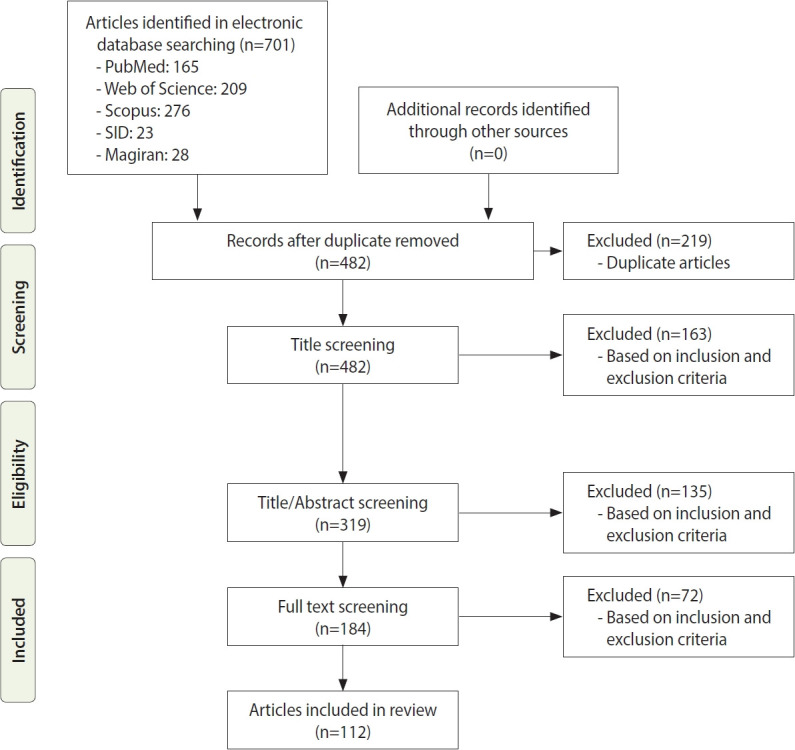
Flow diagram of included/excluded studies.

Figure 1 shows the search strategy and the algorithm of study selection were shown. According to the keywords and Medical Subject Heading terms, 701 studies were identified, of which 163 articles were extracted from the PubMed database, 209 articles from Web of Science, 276 articles from Scopus, 23 articles from SID, and 28 articles from Magiran. Out of these articles, 219 were duplicates that were excluded in the first stage. After identifying relevant studies and considering the inclusion and exclusion criteria, 163 studies, 135 studies, and 72 studies were excluded after screening their titles, abstracts, and full texts, respectively. The studies were reviewed based on the 4-step process of Preferred Reporting Items for Systematic reviews and Meta-Analyses (PRISMA) 2009 (Figure 1), including identifying articles, screening, reviewing the criteria for accepting articles, and determining the articles that entered the meta-analysis process. Finally, 112 articles were included in the final analysis; their information is given in Supplementary Material 1. Of these studies, 66 were performed in Iran; 14 in Egypt; 5 each in Saudi Arabia and Tunisia; 4 each in Pakistan, Iraq, and Afghanistan; 3 each in the United Arab Emirates and Jordan; and 1 each in Sudan, Oman, Somalia, and Lebanon. All articles that were finally included in this study were cross-sectional. We performed a quality assessment for human studies only because the NOS tool is defined for human studies. Nineteen of the 35 (54.2%) included studies had NOS scores of 5-7, indicating that they had high levels of quality, and 12 of the 35 (34.3%) included studies had NOS scores of 3-4, indicating moderate levels of quality (Supplementary Material 1). There was a limited number 4 of the 35 (11.4%) of low-quality studies.

### Publication bias

The results of statistical testing for publication bias, including Begg and Egger tests, for Q fever, were statistically significant for animal milk and blood samples (all p< 0.001). In addition, the result of the Begg test for Q fever in humans was statistically significant (p= 0.01), whereas the result of the Egger test for osteopenia of the lumbar spine was not statistically significant (p= 0.59). Furthermore, the results of the Egger and Begg tests for Q fever in humans were not statistically significant (p= 0.42 and 0.08, respectively). These results confirmed the presence of publication bias.

### The pooled prevalence of Q fever

#### Overall and subgroup

Of the 112 studies that were included in our meta-analysis, most of the studies were conducted on humans and in Iran. The overall prevalence of Q fever in the EMR in humans, animal species, mites and ticks, and milk samples was 22.4% (95% CI, 19.8 to 25.1) and the total heterogeneity was I^2^ = 98.7 (Table 1). According to Table 1, most studies were from 2010 onwards, and studies of Q fever have been conducted in most EMR countries since 2010. Due to the heterogeneity of the selected studies, a random-effects model was used to combine the reported results of the studies.

### Occupation

In human studies, the subjects’ occupations were included in the studies; most studies were conducted among high-risk subjects (butchers, farmers, and veterinarians). However, a limited number of studies have examined other occupations, such as vaccinators, the military, and laboratory occupations. The prevalence estimates of Q fever in the general population, butchers, veterinarians, farmers, herders, military, and other categories were 28.1% (95% CI, 18.0 to 38.3), 31.9% (95% CI, 15.9 to 48.0), 15.6% (95% CI, 2.0 to 29.1), 22.5% (95% CI, 10.5 to 34.4), 62.4% (95% CI, 18.4 to 88.0), 20.3% (95% CI, 2.3 to 38.4), 21.0% (95% CI, 0.0 to 42.5), respectively. The heterogeneity of all occupations was above 86% (Table 1).

### Country

The meta-analysis of studies evaluating Afghanistan (n = 4) showed that the prevalence of Q fever was 34.4% (95% CI, 0.0 to 76.8) (Table 1) with a high level of heterogeneity (99.9 %). The prevalence estimates of Q fever in Egypt, Iran, Iraq, Jordan, Lebanon, Oman, Pakistan, Saudi Arabia, Somalia, Sudan, Tunisia, and the United Arab Emirates were 25.6% (95% CI, 19.0 to 32.3), 20.8% (95% CI, 17.3 to 24.3), 22.2% (95% CI, 0.0 to 44.4), 44.4% (95% CI, 29.6 to 59.1), 20.2% (95% CI, 9.7 to 30.6), 30.0% (95% CI, 0.0 to 71.9), 22.3% (95% CI, 12.4 to 32.2), 20.4% (95% CI, 12.5 to 28.3), 59.1% (95% CI, 52.8 to 65.3), 23.7% (95% CI, 19.8 to 27.6), 15.8% (95% CI, 1.3 to 30.4), and 27.0% (95% CI, 3.5 to 50.5), , respectively. The heterogeneity of all countries was also above 96% (Table 1).

### Species

#### Ticks

**Figure 2.**
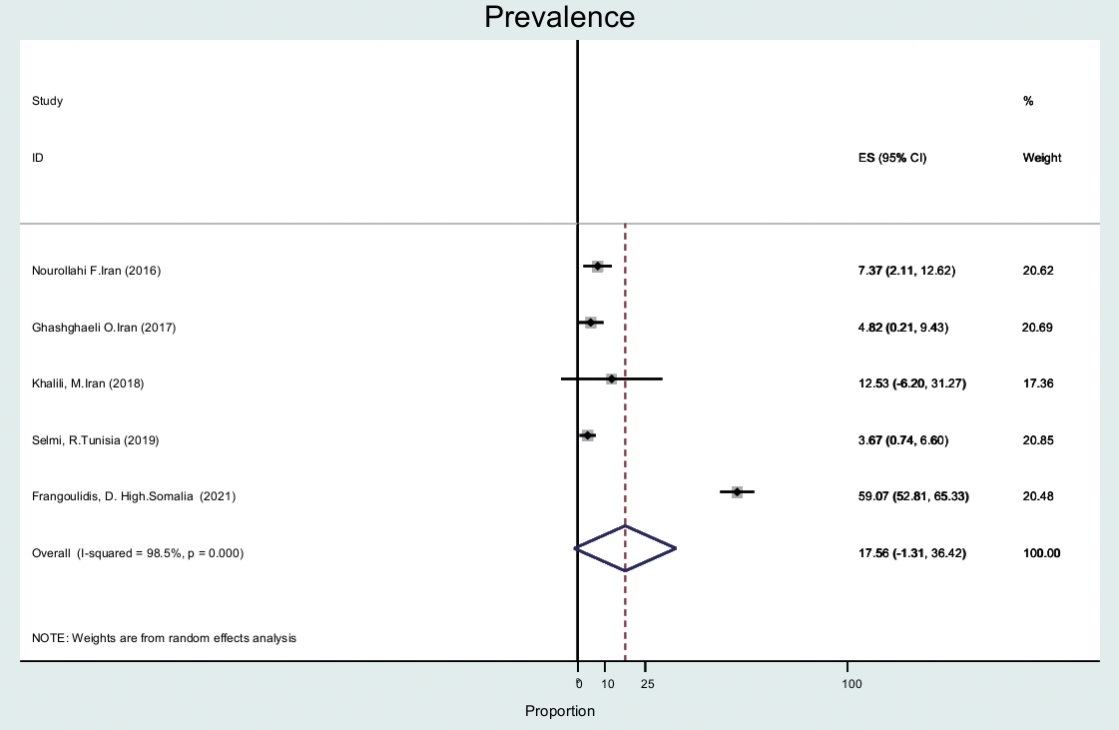
Prevalence of Q fever in ticks in the Eastern Mediterranean region based on a random-effects model. ES, effect size; CI, confidence interval.

The prevalence of Q fever in ticks (n= 5) was 17.5% (95% CI, -1.3 to 36.4), with a high level of heterogeneity (98.5%) (Figure 2). Ticks and mites were collected from animals such as camels, dogs, and small ruminants [24-26].

#### Humans

**Figure 3.**
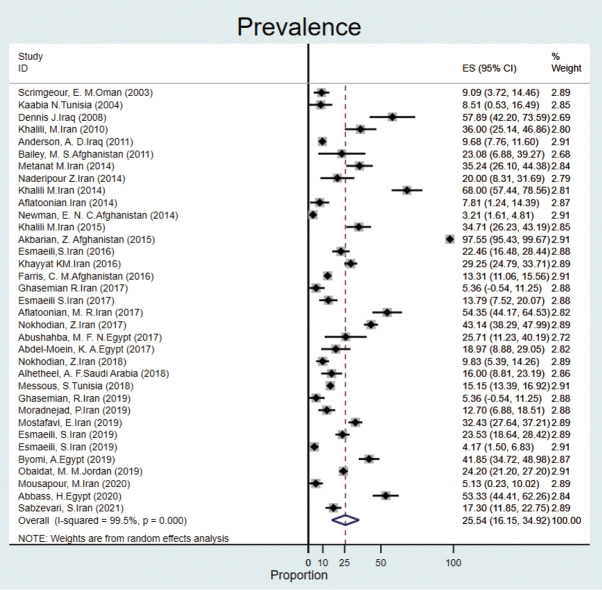
Prevalence of Q fever in humans in the Eastern Mediterranean region based on a random-effects model. ES, effect size; CI, confidence interval.

The prevalence of Q fever in human blood samples was estimated to be 25.5% (95% CI, 16.1 to 34.9), with high heterogeneity (I^2^ = 99.5%) (Figure 3).

#### Animal blood

In animals, milk and blood samples were collected from different species. Eighty out of 92 studies were related to goats, sheep, camels, and cattle, with wide variations in prevalence.

**Figure 4.**
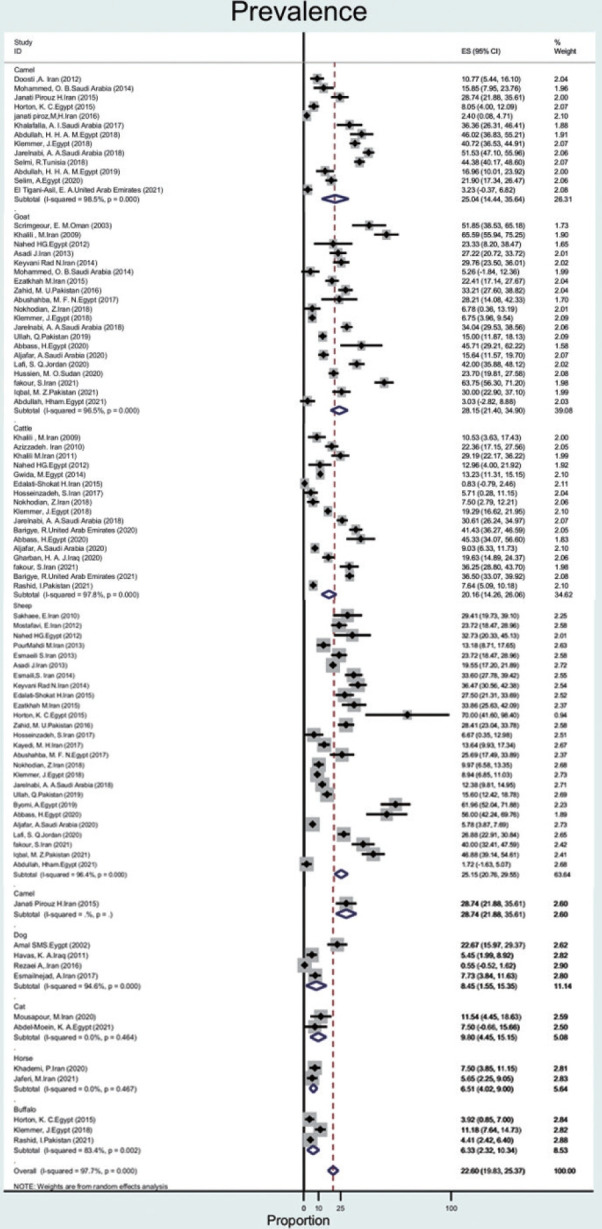
Prevalence of Q fever in animal blood samples from the Eastern Mediterranean region based on a random-effects model. ES, effect size; CI, confidence interval.

The prevalence of Q fever in blood samples from cattle (n= 17) was 20.1% (95% CI, 14.2 to 26.0), with a high level of heterogeneity (97.8%). In goats, sheep, camels, dogs, cats, buffalo, and horses, the prevalence of Q fever was 28.1% (95% CI, 21.4 to 34.9), 25.1% (95% CI, 20.7 to 29.5), 25.0% (95% CI, 14.4 to 35.6), 8.4% (95% CI, 1.5 to 15.3), 9.8% (95% CI, 4.4 to 15.1), 6.3% (95% CI, 2.3 to 10.3), 6.5% (95% CI, 4.0 to 9.0), with heterogeneity of 96.5%, 96.4%, 98.5%,94.6%, not applicable, 83.4%, and not applicable, respectively (Figure 4).

#### Animal milk

**Figure 5.**
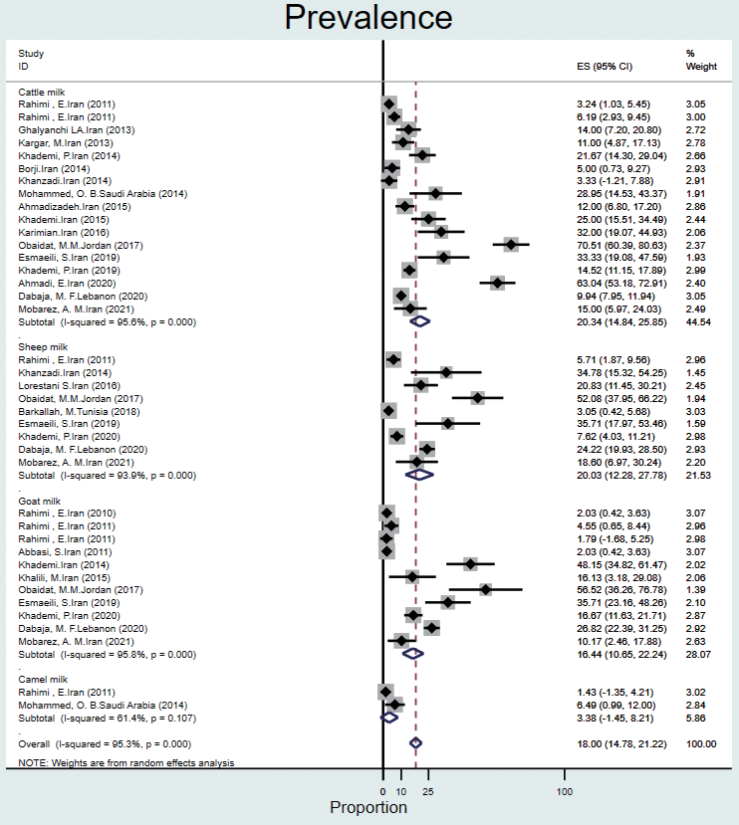
Prevalence of Q fever in animal milk samples from the Eastern Mediterranean region based on a random-effects model. ES, effect size; CI, confidence interval.

The prevalence of Q fever in milk samples from cattle (n= 17) was 20.3% (95% CI, 14.8 to 25.8), and high heterogeneity was observed (95.6%). In sheep, goat, and camel milk samples, the prevalence of Q fever was 20.0% (95% CI, 12.2 to 27.7), 16.4% (95% CI, 10.6 to 22.2), 3.3% (95% CI, -1.4 to 8.2), and the heterogeneity was 93.9%, 95.8%, and 61.4%, respectively (Figure 5).

### Meta-regression

The heterogeneity across studies was particularly high when studies were evaluated overall. For this reason, we performed subgroup analyses and meta-regression. In the subgroup analyses, the I^2^ statistics ranged from 61.4% to 99.5% (Table 1). The meta-regression results showed that species significantly affected the estimation of point prevalence (p= 0.04) (Table 2). That is, studies on certain species showed an excessively high prevalence of Q fever. However, this result does not fully explain the high level of heterogeneity observed (Table 2).

## DISCUSSION

Based on data from 112 studies in the EMR during 2000-2021, we found that Q fever is relatively common among humans, animals, and ticks, with a pooled estimate of 22.4%. The prevalence of Q fever among ticks was 17.5% (95% CI, -1.3 to 36.4). The prevalence of Q fever in blood samples from humans was 25.5% (95% CI, 16.1 to 34.9), that for cattle was 20.1% (95% CI, 14.2 to 26.0), that for goats was 28.1% (95% CI, 21.4 to 34.9), that for sheep was 25.1% (95% CI, 20.7 to 29.5), that for camels was 25.0% (95% CI, 14.4 to 35.6), that for dogs was 8.4% (95% CI, 1.5 to 15.3), that for cats was 9.8% (95% CI, 4.4 to 15.1), that for buffalo was 6.3% (95% CI, 2.3 to 10.3) and that for horses was 6.5% (95% CI, 4.0 to 9.0). The prevalence of Q fever in milk samples from cattle was 20.3% (95% CI, 14.8 to 25.8), that for sheep was 20.0% (95% CI, 12.2 to 27.7), that for goats was 16.4% (95% CI, 10.6 to 22.2), and that for camels was 3.3% (95% CI, -1.4 to 8.2).

However, data on Q fever prevalence were only reported for 14 countries of the region, and there is still a dearth of data across the region, which might be a result of limited resources. Most of the studies were conducted in Iran, Egypt, and Saudi Arabia, and more than half of the studies were carried out in the past 5 years. The results of a review on hotspots of Q fever research from 1990 to 2019, showed that Iran had a total number of publications of 46 (ranked 15th) and was a productive country, with 11 articles in 2019 [27].

The overall seroprevalence in humans was reported in a wide range of countries in the EMR. The results of a systematic review in Iran from 2000 to 2015 revealed that seroprevalence of Q fever in humans was different in various locations; for instance, immunoglobulin G antibodies were reported in 68% and 27.83% of Iranians at high risk in the southeast and western regions, respectively [28]. In our study, the prevalence of Q fever in humans was about 26%, which is similar to the prevalence in the western region of Iran. In the EMR, the highest prevalence of antibodies was seen in Afghanistan (2015), where serological evidence of exposure to 97% of humans (from 204 blood samples) were seropositive for *C. burnetii*, and the lowest (5.12%) was reported in Iran [29,30]. Furthermore, in Iran, 14 epidemiological studies on the distribution of Q fever in humans were conducted between 2016 and 2021. In Egypt, 2 studies (2019-2020) reported that the prevalence of Q fever in humans was 41.8% and 53.3%, respectively [11-25]. The results of studies in Saudi Arabia (2018), Oman (2003), and Tunisia (2004) showed that *C. burnetii* antibodies were detected in 16.0%, 9.8%, and 8.5% of individuals, respectively. In Iraq, the overall percentage of people who had seroconverted to Q fever was 10% (from 909 serum samples). Furthermore, the prevalence of acute Q fever was reported in 5 studies (4 from Iran and 1 from Afghanistan), with a range of 5.3% to 35.2% [31-34]. Therefore, the prevalence of Q fever varies from country to country and even from city to city. Humans can become infected by exposure, most often through inhalation of airborne particles, animal feces, or the release of *C. burnetii* into the environment through feces [35]. Q fever prevalence studies showed evidence of *C. burnetii* prevalence ranging from 15.6% to 62.0% in high-risk populations, including veterinarians, butchers, and farmers [36- 38]. Therefore, this disease can be classified as an occupational disease [39]. Furthermore, the frequency of livestock breeding and the infrastructure of microbiological diagnostic tests, specific epidemiological care, and awareness of the disease affect the spread or control of this disease [35]. Although the prevalence of Q fever may be underestimated, as the symptoms are non-specific and similar to those of other infectious diseases [32], several studies on human antibodies to *C. burnetii* antigens show that Q fever is a major challenge in many countries [40].

Ticks, as main vectors, play an important role in Q fever transmission. The results of our study were slightly different from those of a meta-analysis that was conducted on rodent parasites in the Middle East. In our study, tick prevalence was 18%, compared to 25% in the Middle East [41]. This small difference may be explained by a different context. A systematic study conducted in Europe [42] found the prevalence of Q fever in ticks was 5%, which was different from the prevalence in our study because the prevalence of Q fever in ticks in European countries is lower than in the EMR and this indicates the difference in abundance of species in 2 different regions. This reason may explain the difference in prevalence.

The prevalence of *C. burnetii* in specimens of hard-bodied ticks was only reported in Iran, Somalia, and Tunisia. The results showed that 140 of 237 (59.1%) tick samples in Somalia were positive. In Iran and Tunisia, tick samples were positive in a range of 3% to 17% [43-46] and the tick genera included Rhipicephalus and Hyalomma. In our study, tick samples were obtained from animals including dogs, camels, and small ruminants.

In the region, goats (28%), sheep (25%), and camels (25%) seem to pose the greatest risk for human infection. The results of our study are consistent with a study in Iran that investigated the prevalence of Q fever in animals in 2018. In this study, the total prevalence of Q fever in animals such as cattle, sheep, and goats in Iran was found to be 27%, of which the highest prevalence was 33% for goats. In our study, the prevalence of Q fever in goats (28%) than that of other animals [28]. Furthermore, in line with our results, in the United States, goats (42%) and sheep (16%) appear to pose a greater risk of human infection than cattle or wild animals [3]. The *C. burnetii* seroprevalence in camel herds can reach more than 60% in Egypt, Saudi Arabia, and Sudan [47]. Although the prevalence of *C. burnetii* in ruminants was over 20%, it was less than 10% in dogs, cats, camels, and horses. Therefore, this disease poses a major risk for the contamination of ruminants.

In milk samples, the highest prevalence was reported in goats (48%) and sheep (21%) in Iran [48,49]. In a study in the United States [50], the prevalence of Q fever in bulk tank milk of dairy herds was ≥ 94%. The high prevalence of *C. burnetii* in dairy herds can reflect the lack of effective vaccines and treatment protocols for infected animals [50]. In a meta-analysis conducted in Iran [51], the total prevalence of *C. burnetii* was 15.09% in cattle milk, 7.80% in goat milk, and 3.79% in sheep milk. This disease has become a special challenge in a system with mixed herds of cattle, sheep, and goats, and it is considered one of the important problems of livestock production all over the world [52].

According to One Health goals, monitoring and surveillance of Q fever in livestock, wildlife, and humans is crucial in identifying and controlling outbreaks [53]. Furthermore, controlling external parasites such as ticks should be considered to reduce their adverse effects [54].

The most important strength of the present study is the comprehensive review of all databases, the independent review of articles by 2 researchers, and the performance of meta-regression and subgroup analyses to obtain more accurate information. The present study aimed to address the limitations of other systematic reviews and meta-analyses in this field by conducting a comprehensive review of different sources over a long period, with meta-regression and subgroup analyses. However, the wide variety of sampling, testing, and recording methods in different studies and the relatively long period of 21 years may reduce the comparability of the findings.

## CONCLUSION

Our study findings demonstrate that Q fever is a public health issue in the EMR. For an effective zoonotic disease surveillance program in the region, additional research on human and animal species in areas where there is a lack of knowledge about the distribution of Q fever is necessary, in addition to interventions for prevention and control.

## Figures and Tables

**Table 1.** Prevalence of Q fever according to various items

Subgroup	No. of articles	I^2^ (%)	Prevalence, % (95% CI)
Year			
2000-2009	7	97.3	31.8 (13.1, 50.4)
2010-2015	58	98.9	21.0 (16.2, 25.7)
+2016	101	98.7	23.9 (20.5, 27.3)
Country			
Afghanistan	4	99.9	34.4 (0.0, 76.8)
Egypt	29	98.2	25.6 (19.0, 32.3)
Iran	87	98.4	20.8 (17.3, 24.3)
Iraq	4	99.2	22.2 (0.0, 44.4)
Jordan	6	97.0	44.4 (29.6, 59.1)
Lebanon	3	96.2	20.2 (9.7, 30.6)
Oman	2	97.0	30.0 (0.0, 71.9)
Pakistan	8	98.3	22.3 (12.4, 32.2)
Saudi Arabia	13	97.9	20.4 (12.5, 28.3)
Somalia	1	-	59.1 (52.8, 65.3)
Sudan	1	-	23.7 (19.8, 27.6)
Tunisia	5	99.1	15.8 (1.3, 30.4)
United Arab Emirates	3	99.0	27.0 (3.5, 50.5)
Occupation			
General	19	98.8	28.1 (18.0, 38.3)
Butcher	7	98.3	31.9 (15.9, 48.0)
Veterinarian	4	87.7	15.6 (2.0, 29.1)
Farmer	5	86.1	22.5 (10.5, 34.4)
Herder	2	-	62.4 (18.4, 88.0)
Military	5	99.5	20.3 (2.3, 38.4)
Other	2	96.1	21.0 (0.0, 42.5)
Species			
Buffalo	3	83.4	6.3 (2.3, 10.3)
Camels	13	98.5	25.0 (14.4, 35.6)
Camel milk	2	61.4	3.3 (-1.4, 8.2)
Cats	2	-	9.8 (4.4, 15.1)
Cattle	17	97.8	20.1 (14.2, 26.0)
Cattle milk	17	95.6	20.3 (14.8, 25.8)
Dogs	4	94.6	8.4 (1.5, 15.3)
Goats	20	96.5	28.1 (21.4, 34.9)
Goat milk	11	95.8	16.4 (10.6, 22.2)
Horses	2	-	6.5 (4.0, 9.0)
Humans	34	99.5	25.5 (16.1, 34.9)
Sheep	26	96.4	25.1 (20.7, 29.5)
Sheep milk	9	93.9	20.0 (12.2, 27.7)
Ticks	5	98.5	17.5 (-1.3, 36.4)
Total		98.7	22.4 (19.8, 25.1)

**Table 2.** Results of Meta-regression for the prevalence of Q fever

Covariate	Meta-regression coefficient	95% CI		p-value
		LL	UL	
Year of publication	0.1	-4.7	5.0	0.95
Country	-0.1	-0.8	0.6	0.75
Species	0.1	0.0	0.2	0.04
Occupation	-0.2	-1.5	1.0	0.70

CI, confidence interval; LL, lower limit; UL, upper limit.

## SUPPLEMENTARY MATERIALS

Supplementary Material 1. Included in the final analysis; their information
