# Supplementary material for: The prevalence of Q fever in the Eastern Mediterranean region: a systematic review and meta-analysis
Source: Epidemiol Health. 2022 Oct 28;44:e2022097. doi: 10.4178/epih.e2022097 (PMC10396516; doi:10.4178/epih.e2022097)
Supplement: Supplementary Material 1. — Included in the final analysis; their information [file epih-44-e2022097-Supplementary-1.docx]

**Supplementary Material 1. Included in the final analysis; their information**

| Disease/Pathogen | YEAR | Country | design | population | Number of Animals/Humans/Samples Tested | Study Outcome/Disease Frequency/Seroprevalence | Reference | Score study human |
| --- | --- | --- | --- | --- | --- | --- | --- | --- |
| Q fever/ C. burnetii | 2002 | Egypt | Cross-sectional | dog | 150 serum sample | In total, 34 of 150 (23%) serum sample dogs were positive. | Amal SMS^1^ |  |
| Q fever/ C. burnetii | 2003 | Oman | Cross sectional | humans and goat | 110 blood sample humans  54 blood sample goats | The human revealed that 10 (9.8%) were seropositive for previous Coxiella burnetii  The goat revealed that 28 (52%) had been infected Coxiella burnetii | Scrimgeour, E. M.^2^ | 4 |
| Q fever/ C. burnetii | 2004 | Tunisia | Cross-sectional | human | 47 serum sample | In total, 4 of 47 (8.5%) serum sample human were positive. | Kaabia N^3^ | 2 |
| Q fever/ C. burnetii | 2008 | Iraq | Cross-sectional | human | 38 serum sample | An outbreak of Q fever occurred in 22 (58%) of 38 Marines deployed to Iraq in 2005 | Dennis J.^4^ | 4 |
| Q fever/ C. burnetii | 2009 | Iran | Cross sectional | goat and cattle | 169 serum sample (76 goat samples and 93 cattle samples) | The seroprevalence of Q fever were 35.5% (N = 60). Goats (65.78%) and cattle (10.75%) | Khalili, M.^5^ |  |
| Q fever/ C. burnetii | 2010 | Iran | Cross sectional | sheep | 85 serum sample | Antibodies were detected in 25 sera (29.42%) of 85 samples. | Sakhaee, E. ^6^ |  |
| Q fever/ C. burnetii | 2010 | Iran | Cross sectional | cattle | 246 | Seroprevalence of Coxiella burnetii 22.3% of 246 dairy cattle in Khorasan Razavi | Azizzadeh,M^7^ |  |
| Q fever/ C. burnetii | 2010 | Iran | Cross sectional | goat | 296 milk samples | total, 6 of 296 (2.0%) goat milk samples were positive | Rahimi,E^8^ |  |
| Q fever/ C. burnetii | 2010 | Iran | Cross sectional | human | 75 serum sample | Iran phase II antibodies in 27 subjects (36%) | Khalili, M^9^ | 3 |
| Q fever/ C. burnetii | 2011 | Iran | Cross sectional | cattle, sheep, camel, goat | 567 milk samples (247 cattle, 140 sheep, 70 camel, 110 goat) | In total, 8 of 247 (3.2%) cattle milk. 8 of 140 (5.7%) sheep bulk milk and 5 of 110 (4.5%) goat bulk and One of 70 (1.4%) camel bulk milk was also positive for C. burnetii. | Rahimi E^10^ |  |
| Q fever/ C. burnetii | 2011 | Iran | Cross sectional | cattle, goat | 320 milk samples (210 cattle, 110 sheep,56 goat) | 13 of 210 (6.2%) of cattle bulk milk samples, 1 of 56 (1.8%) of goat bulk milk samples and 0% of sheep bulk milk samples were positive for C. burnetiid. | Rahimi, E.^11^ |  |
| Q fever/ C. burnetii | 2011 | Iran | Cross sectional | cattle | 161 serum sample | The sera of cattle with reproductive problems showed ahigh prevalence (51.35%) of anti-C.burnetii antibodies. Bycontrast, the sera of apparently healthy cattle showed arelatively low prevalence (10.3%) of anti-C.burnetii anti-bodies | Khalili M ^12^ |  |
| Q fever/ C. burnetii | 2011 | Iran | Cross sectional | goat | 296 milk sample | six out of 296 (2.0%) goat milk samples were positive; | Abbasi, S.^13^ |  |
| Q fever/ C. burnetii | 2011 | Iraq | Cross-Sectional | Military working dog, wild canine | 280(165 feral canin and 115 Military working dogs) | None of the 115 MWDs tested were seropositive for disease. Nine of the 165 indigenous canines were seropositive resulting in a prevalence of 5.5% (95% CI: 2.5%–10.1%) | Havas, K. A.^14^ |  |
| Q fever/ C. burnetii | 2011 | Iraq | Cross sectional | human | 909 serum samples | The overall number who seroconverted to Q fever was 88 (10%) | Anderson, A. D.^15^ | 4 |
| Q fever/ C. burnetii | 2011 | Afghanistan | Cross sectional | human | 26 blood sample | Over 6 months, there were 26 cases of “Helmand Fever” assessed and 6 (26%) were acute Q fever | Bailey, M. S.^16^ | 3 |
| Q fever/ C. burnetii | 2012 | Iran | Cross sectional | camel | 130 Blood samples | 14 (10.76 %) camel blood samples were found PCR positive | Doosti, A. ^17^ |  |
| Q fever/ C. burnetii | 2012 | Iran | Cross sectional | sheep | 253 sera | The infection rate with Q fever was 23.7% | Mostafavi,E^18^ |  |
| Q fever/ C. burnetii | 2012 | Egypt | Cross-sectional | sheep, goat, cattle and buffaloes, | 184 serum sample (55 sheep, 30 goats, 54 cattle and 45 buffaloes ( | The overall seroprevalence in ruminants was 17.4%while displayed in different species as (32.7%, 23.3%, and 13%) for sheep, goats and cattle respectively whereas none of examined buffaloes was positive | Nahed HG^19^ |  |
| Q fever/ C. burnetii | 2013 | Iran | Cross sectional | sheep | 220 Serum samples | Seroprevalence of Q fever was 13.18% | PourMahdi M^20^ |  |
| Q fever/ C. burnetii | 2013 | Iran | Cross sectional | sheep | 253 sera | The seroconversion rate was 23.7%. | Esmaeili S^21^ |  |
| Q fever/ C. burnetii | 2013 | Iran | Cross sectional | sheep and goat | 1280 serum sample (110 sheep and 280 goat) | seroprevalence of C. burnetii in sheep and goats was 19.5% and 27.2%. | Asadi J^22^ |  |
| Q fever/ C. burnetii | 2013 | Iran | Cross sectional | cattle | 100 milk sample | In this study, 14% (14 of 100) of bulk milk was positive | Ghalyanchi LA^23^ |  |
| Q fever/ C. burnetii | 2013 | Iran | Cross sectional | cattle | 100 milk samples | Of the tested samples, 11% tested positive for C. burnetii | Kargar, M.^24^ |  |
| Q fever/ C. burnetii | 2014 | Iran | Cross sectional | sheep | 253 sera | A total of 33.6% of sheep sera and 87.50% of herds were positive for C. burnetii IgG | Esmaeili S ^25^ |  |
| Q fever/ C. burnetii | 2014 | Egypt | Cross sectional | cattle | 1,194 blood sera | 158 cows (13.2%) had anti-Coxiella antibodies. | Gwida, M.^26^ |  |
| Q fever/ C. burnetii | 2014 | Iran | Cross sectional | cattle | 100milk samples | Five samples (5%) of catttle milk were found positive for C. burnetii. | Borji^27^ |  |
| Q fever/ C. burnetii | 2014 | Saudi Arabia | Cross sectional | camel, goat, cattle | 235 samples including blood, milk (camel milk 77, camel blood82, goat blood 38, cattle Milk 38) | A percentage of 10.8% samples yielded positive PCR amplification from both blood and milk, where 15 of 139 blood and 16 of 148 milk samples were positive. | Mohammed, O. B.^28^ |  |
| Q fever/ C. burnetii | 2014 | Iran | Cross sectional | cattle, sheep | 23 sheep milk samples, and 60 cattle milk samples, | 8 of 23 (34.78%) sheep milk samples, and 2 of 60 (3.33%) cow milk samples were found to be positive for C. burnetiid. | Khanzadi,S^29^ |  |
| Q fever/ C. burnetii | 2014 | Iran | Cross sectional | goat | 54milk samples | total of 54 goat milk samples, 26 samples (48%) were found to be positive for the presence of Coxiella burnetii. | Khademi^30^ |  |
| Q fever/ C. burnetii | 2014 | Iran | Cross sectional | Sheep, goat | 460 sera (Sheep 255 and goats 205) | Seroprevalence of C. burnetii at animal level was 36.5% (95% CI: 30.6%-42.4%) for sheep and 29.8% (95% CI: 23.8%-36.2%) for goat populations | Keyvani Rad N^31^ |  |
| Q fever/ C. burnetii | 2014 | Afghanistan | Cross sectional | human | 467 serum samples | Among the samples tested, 15 samples (3.2%) were positive for Coxiella burnetii | Newman,E,A^32^ | 5 |
| Q fever/ C. burnetii | 2014 | Iran | Cross-sectional | cattle | 120 milk samples | In this study, in total, 26 samples (21.66%) were found to be positive for the presence of Coxiella burnetii | Khademi, P.^33^ |  |
| Q fever/ C. burnetii | 2014 | Iran | Cross sectional | human | 105 serum sample | Among 105 patients 35.2% (37/105) febrile patients had a positive serology test for acute Q fever. | Metanat M. ^34^ | 5 |
| Q fever/ C. burnetii | 2014 | Iran | Cross sectional | human | 45 serum samples | this kit revealed 9 sera (20 %) as positive | Naderipour Z.^35^ | 5 |
| Q fever/ C. burnetii | 2014 | Iran | Cross-sectional | human | 64 serum samples | Among all sera samples tested, only 5 samples (7.8%) were positive for the presence of IgM antibodies | Aflatoonian^36^ | 2 |
| Q fever/ C. burnetii | 2014 | Iran | Cross sectional | human | 75 serum sample | The positive rate of IgG antibody was 68% in the slaughterhouse workers. | Khalili M^37^ | 5 |
| Q fever/ C. burnetii | 2015 | Iran | Cross sectional | human | 121 blood samples | Results showed that 34.7% were positive from all the serum samples. | Khalili M^38^ | 3 |
| Q fever/ C. burnetii | 2015 | Iran | Cross sectional | sheep, goat and cattle | 370 serum sample (200 sheep, 50 goats and 120 dairy cattle) | Antibodies to C. burnetii were found in 27.5% of sheep, 54% in goats and 0.83% in dairy cattle | Edalati-Shokat H^39^ |  |
| Q fever/ C. burnetii | 2015 | Iran | Cross sectional | goat | 31 milk sample | In total, 5 of 31 (16.12%) goat milk samples were positive | Khalili, M.^40^ |  |
| Q fever/ C. burnetii | 2015 | Iran | Cross sectional | cattle | 150milk samples | After the PCR test on 150 simples’ number of milk, 18 positive samples were obtained which confirms to 12% of existence of bacteria in milk samples | Ahmadizadeh^41^ |  |
| Q fever/ C. burnetii | 2015 | Iran | Cross sectional | camel | 167 sera | Seroprevalence of C. burnetii at animal level was 28.7 % | Janati Pirouz H^42^ |  |
| Q fever/ C. burnetii | 2015 | Iran | Cross sectional | goat and sheep | 368 blood samples (241 goat and 127 sheep) | Seropositivity 97 animals (26.4%), including 43 sheep (33.9%) and 54 goats (22.4%), had antibodies to C. burnetiid | Ezatkhah M^43^ |  |
| Q fever/ C. burnetii | 2015 | Afghanistan | Cross sectional | human | 204 Blood samples | 97% of humans had at least one C. burnetii seropositive person | Akbarian, Z.^44^ | 5 |
| Q fever/ C. burnetii | 2015 | Iran | Cross sectional | cattle | 80 milk samples | In this study, 20 out of 80 milk samples (25%) were  positive in terms of Coxiella burnetii. | Khademi^45^ |  |
| Q fever/ C. burnetii | 2015 | Egypt | ELISA | Buffalo, sheep and camels; | 337 blood sample Buffalo (n = 153)  Sheep (n = 174)  Camels (n = 10) | antibodies against C. burnetii in six (4%) buffalo, 14 (8%) sheep, and seven (70%) camels; | Horton, K. C^46^. |  |
| Q fever/ C. burnetii | 2016 | Iran | Cross sectional | Ticks | 1305 | Prevalence of ixodid tick infestation in small ruminants was 58.4% | Nourollahi F.^47^ |  |
| Q fever/ C. burnetii | 2016 | Pakistan | Cross sectional | sheep and goats | 542 sera sample (271 sheep and goat271) | A high herd level prevalence (73.1%, 95% CI 63.5-81.3) was recorded in the studied districts. Individual level seroprevalence was recorded as 30.8% (95% CI 26.9-34.9) | Zahid, M. U.^48^ |  |
| Q fever/ C. burnetii | 2016 | Iran | Cross-sectional | human | 400 serum sample | The overall prevalence of C. burnetii in sera from pregnant women was 29.3% (95% confidence interval (CI): 25-34%). | Khayyat KM^49^ | 6 |
| Q fever/ C. burnetii | 2016 | Iran | Cross sectional | camel | 167 blood samples | 4 of 167 camels blood samples positive 2.4 %. | janati piroz,M,H ^50^ |  |
| Q fever/ C. burnetii | 2016 | Iran | Cross sectional | human | 190 blood samples | The seroprevalence of Q fever were 22.5%. | Esmaeili,S. ^51^ | 7 |
| Q fever/ C. burnetii | 2016 | Iran | Cross sectional | dog | 182 serum sample | Seroprevalence of both Q fever and Lyme disease was 0.55% (95% CI: 0-2.7%) | Rezaei A^52^ |  |
| Q fever/ C. burnetii | 2016 | Afghanistan | Cross sectional | human | 879 serum samples | Analysis samples showed that 117 (13.3) % of the seroconverted for the Q fever. | Farris, C. M.^53^ | 4 |
| Q fever/ C. burnetii | 2016 | Iran | Cross sectional | cattle | 50milk samples | In this survey, 16 out of 50 (32%) cow bulk tank milk samples were positive for presence of Coxiella burnetii | Karimian^54^ |  |
| Q fever/ C. burnetii | 2016 | Iran | Cross sectional | Sheep | 72milk samples | number 15 out of 72 (20.83%) sheep milk samples were positive for C. burnetii | Lorestani S^55^ |  |
| Q fever/ C. burnetii | 2017 | Egypt | Cross-sectional | Sheep, goat, human | 183 serums (109 sheep,39 goat,35 humans) | The seroprevalence of C. burnetii IgG antibodies was 25.68% (28 of 109), 28.20% (11 of 39) and 25.71% (9 of 35) in sheep, goats, and humans | Abushahba, M. F. N.^56^ | 5 |
| Q fever/ C. burnetii | 2017 | Iran | Cross sectional | Ticks | 583 ticks | The overall prevalence of hard tick infestation on cattle was 56.8% | Ghashghaeli O^57^ |  |
| Q fever/ C. burnetii | 2017 | Egypt | Cross-Sectional | human | 58 humans | the seroprevalence of C. burnetii IgG antibodies among human was (11/58) 19%. | Abdel-Moein, K. A.^58^ | 4 |
| Q fever/ C. burnetii | 2017 | Saudi Arabia | Cross sectional | camel | 88 Serum samples | Coxiella burnetii 32/88 (37%) | Khalafalla, A. I.^59^ |  |
| Q fever/ C. burnetii | 2017 | Jordan | Cross sectional | Cattle, Sheep, and Goats | 149 milk sample (78 cattle, 48 sheep, and 23 goats) | Positive results were obtained from 70.9% (60.6 to 79.5%) of dairy cattle, 52.1% (38.3 to 65.5%) of sheep, and 56.0% (37.1 to 73.3%) of goat | Obaidat, M.M^60^ |  |
| Q fever/ C. burnetii | 2017 | Iran | Cross sectional | sheep and cattle | 130 serum samples (60 sheep and 70 cattle ( | The results showed that 6.66% of the sheep and 5.71% of the cattle carcasses were positive | Hosseinzadeh, S.^61^ |  |
| Q fever/ C. burnetii | 2017 | Iran | Cross sectional | human | 56 blood samples | The prevalence rate of acute Q fever in 56 patients with 2nd blood sample was 5.3% | Ghasemian R^62^ | 4 |
| Q fever/ C. burnetii | 2017 | Iran | Cross sectional | human | 92 blood sample | The results showed that 50 serum samples (54.35%) were positive | Aflatoonian, M. R.^63^ | 6 |
| Q fever/ C. burnetii | 2017 | Iran | Cross sectional | human | 401 serum samples | The overall seropositivity (presence of antibodies against phase I and/or phase II) was 43.1% | Nokhodian, Z^64^. | 6 |
| Q fever/ C. burnetii | 2017 | Iran | Cross sectional | dog | 181 blood sample | Out of the 181 sera samples studied using the ELISA method, 14 (7.7%) had anti-bodies against Coxiella burnetii, | Esmailnejad, A.^65^ |  |
| Q fever/ C. burnetii | 2017 | Iran | Cross sectional | sheep | 330 blood sample | Among the samples tested, 45 samples (13.64%) were seropositive | Kayedi, M. H.^66^ |  |

| Q fever/ C. burnetii | 2017 | Iran | Cross sectional | human | 116 blood sample | The prevalence of acute Q fever was 13.8% (95% confidence interval [CI]: 8.0, 21.0%) | Esmaeili S.^67^ | 6 |
| --- | --- | --- | --- | --- | --- | --- | --- | --- |
| Q fever/ C. burnetii | 2018 | Iran | Cross-sectional | Cattle, sheep, goat | 480 blood samples (120 cattle, 301 sheep,59 goat) | In total, 7.5% of the cattle, 10% of the sheep, and 6.8% of the goats were positive and 25 herds had at least a positive sample | Nokhodian, Z.^68^ |  |
| Q fever/ C. burnetii | 2018 | Iran | Cross sectional | human | 173 blood samples | In total, 9.83% of the samples were positive | Nokhodian, Z.^69^ |  |
| Q fever/ C. burnetii | 2018 | Iran | Cross-sectional | tick | 375 | In total, 47 of 375 (12.5%) tick samples were positive | Khalili, M.^70^ |  |
| Q fever/ C. burnetii | 2018 | Egypt | Cross sectional | camel | 113 serum samples | A total of 52 camels (46%) were positive for Q fever infection | Abdullah, H. H. A. M.^71^ |  |
| Q fever/ C. burnetii | 2018 | Egypt | Cross sectional | Camel, cattle, buffaloes, sheep, goat | 2,699 blood samples (Camels 528, cattle 840, buffaloes 304, sheep 716, goats 311) | Coxiella burnetii specific antibodies were detected in 40.7% of camels (215/528), 19.3% of cattle (162/840), 11.2% of buffaloes (34/304), 8.9% of sheep (64/716) and 6.8% of goats (21/311), respectively. | Klemmer, J.^72^ |  |
| Q fever/ C. burnetii | 2018 | Saudi Arabia | Cross sectional | Camel, cattle, sheep, goats | Serum samples from 489 camels, 428 cattle, 630 sheep and 423 goats | The overall seroprevalence was 30.71%. Prevalence by species was 51.53%, 30.67%, 34.04% and 12.38% in camels, cattle, goats and sheep | Jarelnabi, A. A.^73^ |  |
| Q fever/ C. burnetii | 2018 | Saudi Arabia | Cross sectional | human | 100 blood sample | Coxiella burnetii phase 1 and phase 2 antibodies were detected in16 (16%) of the patients. | Alhetheel, A. F.^74^ |  |
| Q fever/ C. burnetii | 2018 | Tunisia | Cross sectional | sheep | A total of 164 animals: 164 blood, 164 vaginal swabs and 164 milk samples | C. burnetii was detected in 12 (7.31%) vaginal swab, 5 (3.04%) milk and 4 (2.43%) blood samples | Barkallah, M.^75^ |  |
| Q fever/ C. burnetii | 2018 | Tunisia | Cross sectional | human | 240 serum sample | The prevalence of C. burnetii was 6.6%, respectively | Messous, S.^76^ | 3 |
| Q fever/ C. burnetii | 2018 | Tunisia | Cross sectional | camel | 534 serum sample | Overall, 237 camels (44%, 95%CI: 0.40-0.49) were seropositive to C. burnetii | Selmi, R. ^77^ |  |
| Q fever/ C. burnetii | 2019 | Iran | Cross sectional | human | 216 serum samples | 9/216 (4.2%) case was positive for coxiella burnetiid. | Esmaeili, S.^78^ |  |
| Q fever/ C. burnetii | 2019 | Iran | Cross-sectional | human | 476 serum sample | The prevalence of acute Q fever was 5.37% (95% CI: 1.84, 14.61%). | Ghasemian, R.^79^ | 6 |
| Q fever/ C. burnetii | 2019 | Iran | Cross sectional | cattle | 420 milk sample | The results showed that 14.6% cattle samples) were positive for C. burnetii. | Khademi, P.^80^ |  |
| Q fever/ C. burnetii | 2019 | Egypt | Cross sectional | camel | 112 blood sample | Out of 112 camels, 19 were positive for C. burnetii by qPCR with an overall prevalence of 16.9% | Abdullah, H. H. A. M.^81^ |  |
| Q fever/ C. burnetii | 2019 | Egypt | Cross sectional | Sheep, human | 276 serum sample (sheep 92, human 184) | The seroprevalence of anti-C. burnetii phase II IgG antibodies were 61.96% (57/92) among sheep and 41.85% (77/184) among humans by using ELISA | Byomi, A.^82^ |  |
| Q fever/ C. burnetii | 2019 | Tunisia | Cross sectional | Ticks | 327 serum samples | overall prevalence rate of 8% (26/327) | Selmi, R.^83^ |  |
| Q fever/ C. burnetii | 2019 | Iran | Cross sectional | human | 289serum samples | The seroprevalence of Q fever were 23.5%. | Esmaeili, S.^84^ |  |
| Q fever/ C. burnetii | 2019 | Iran | Cross sectional | human | 126 blood sample | Among the participants, 16 patients (30.77%) were diagnosed with Q fever | Moradnejad, P.^85^ |  |
| Q fever/ C. burnetii | 2019 | Jordan | Cross sectional | human | 781 serum sample | The overall seroprevalence for C. burnetii was 24.2% (95% CI; 21.3-27.3%). | Obaidat, M.M ^86^ |  |
| Q fever/ C. burnetii | 2019 | Iran | Cross sectional | human | 367 sera samples | The seroprevalence of antibodies against C. burnetii in 32.42% overall. | Mostafavi, E.^87^ |  |
| Q fever/ C. burnetii | 2019 | Iran | Cross-sectional | cattle, sheep, goat | 126 milk samples (42 cattle, 26 sheep, 56 goat) | In total, 34.92% (44 of 126) milk samples were positive for C. burnetiid. Prevalence of C. burnetii in cattle, sheep and goat milk was 33.33%, 35.71% and 35.71%, respectively. | Esmaeili, S.^88^ |  |
| Q fever/ C. burnetii | 2019 | Pakistan | Cross sectional | Sheep, goat | 1000 serum samples (500 from sheep and 500 from goats) | Total prevalence of (153/1000) 15.3%, prevalence Coxiella burnetii in sheep (78/500) 15.6% and (75/500) 15.0% in goats. | Ullah, Q.^89^ |  |
| Q fever/ C. burnetii | 2020 | United Arab Emirates | Cross sectional | cattle | 350 serum samples | Of the 350 cattle, 41.4%, were seropositive to C. burnetiid. | Barigye, R.^90^ |  |
| Q fever/ C. burnetii | 2020 | Iran | Cross sectional | cattle | 92 milk sample | Further, anti‐C. burnetii antibody was detected in 63.04% of bulk tank milk samples | Ahmadi, E.^91^ |  |
| Q fever/ C. burnetii | 2020 | Iran | Cross sectional | sheep and goat | 420 milk samples (sheep 210 and goats 210) | The total prevalence 51/420 (12.1%) examined samples sheep 16/210 (7.6%) and goat 35/210 (16.6%) were positive for C. burnetii. | Khademi, P.^92^ |  |
| Q fever/ C. burnetii | 2020 | Iran | Cross sectional | horse | 200 serum samples | he results showed that 7.50 % (P < 0.05; 95 % CI: 0.5 %-0.12 %) of the examined sera samples were positive for C. burnetii | Khademi, P.^93^ |  |
| Q fever/ C. burnetii | 2020 | Iran | Cross-sectional | cat and human | 241 serum sample (stray cats 85, domestic cats 78, human 78) | Antibodies were detected in 19 sera of 85 (22.35%) samples in stray cats, 9 sera of 78 (11.53%) samples of domestic cats and 4 sera of 78 (5.12%) samples of their owners. | Mousapour, M.^94^ |  |
| Q fever/ C. burnetii | 2020 | Egypt | Cross sectional | camel | 315 serum sample | seroprevalence of C. burnetii among camels was 22 % | Selim, A.^95^ |  |
| Q fever/ C. burnetii | 2020 | Jordan | Cross sectional | goat, sheep | 730 Serum samples (goat 250, sheep 480) | The overall goat and sheep seroprevalence level was 32.5% (237/730) and was significantly higher in goats (43.3%, 108/250) than sheep (27%, 129/480) | Lafi, S. Q.^96^ |  |
| Q fever/ C. burnetii | 2020 | Sudan | Cross sectional | goat | 460 serum sample | The results showed an overall prevalence rate (109/460) 24.22% of Q fever antibodies | Hussien, M. O.^97^ |  |
| Q fever/ C. burnetii | 2020 | Saudi Arabia | Cross sectional | cattle, sheep, and goat | 1310 serum samples (cattle 432, sheep 571, and goats 307) | The prevalence of C. burnetii infection among animals was 9.2% (CI, 7.7-10.8)-15.6%, 9.1%, and 5.8% among goats, cattle, and sheep | Aljafar, A.^98^ |  |
| Q fever/ C. burnetii | 2020 | Iraq | Cross sectional | cattle | 270 serum samples | a total of (53/270) 19.63% cows were seropositives to C. burnetii IgG antibodies. | Gharban, H. A. J.^99^ |  |
| Q fever/ C. burnetii | 2020 | Lebanon | Cross sectional | ruminants (865 cattle, 384 sheep and 384 goats) | 1633 serum samples | Seroprevalence in cattle, sheep and goat were 9.94% (86/865), 24.2% (93/384) and 26.8% (103/384). | Dabaja, M. F^100^ |  |
| Q fever/ C. burnetii | 2020 | Egypt | Cross-sectional | cattle, sheep, goat, and human | 280 serum sample (cattle 75, sheep 50, goat 35, human120) | The results of the IFA revealed C. burnetii seroprevalence rates of 45.3%, 56.0%, 45.7%, and 53.3% in cattle, sheep, goats, and humans, respectively. | Abbass, H.^101^ | 5 |
| Q fever/ C. burnetii | 2021 | Iran | Cross sectional | goat, sheep, and cattle | 162 milk sample (59 goat, 43 sheep,60 cattle) | In total, 23 of 162 samples were positive for C. burnetii, In goat milk sample 10.17%. In sheep milk samples, 18.6% were positive, and C. burnetii was detected in 15% (95% CI: 8.1–26.11) of cattle milk samples | Mobarez, A. ^102^M. |  |
| Q fever/ C. burnetii | 2021 | Iran | Cross sectional | horse | 177 blood samples and 115 vaginal swabs | Antibodies were detected in 5.64 % (10/177) of sera samples and C. burnetii DNA was detected in 7.82 % (9/115) of horse vaginal samples | Jaferi, M.^103^ |  |
| Q fever/ C. burnetii | 2021 | Iran | ELISA | human | 185 sera sample | The seroprevalence of antibodies against C. burnetii was 17.2% | Sabzevari, S.^104^ |  |
| Q fever/ C. burnetii | 2021 | Iran | Cross sectional | sheep, goat and cattle | 480 blood samples (160 for sheep, goats and cattle) | the seroprevalence of (224/480) 46.6 % for Q fever. Seroprevalence in sheep, goats and cattle were 28.58 % (64/160), 45.53 % (102/160) and 25.89 % (58/160). | fakour, S.^105^ |  |
| Q fever/ C. burnetii | 2021 | United Arab Emirates | Cross sectional | camel | 93 blood samples | DNA investigations on camel blood samples showed a positivity for Coxiella burnetii (3.2%) | El Tigani-Asil, E. A.^106^ |  |
| Q fever/ C. burnetii | 2021 | United Arab Emirates | Cross sectional | cattle | 759 Blood, sera sample | Of the 759 study cattle, 36.5% (277/759) were seropositive | Barigye, R.^107^ |  |
| Q fever/ C. burnetii | 2021 | Pakistan | Cross sectional | sheep and goat | 320 blood samples (sheep 160 and goats 160) | The results showed that the overall positive percentage of C. burnetii is 36.87% (sheep: 46.9% and goats: 30%). | Iqbal, M. Z.^108^ |  |
| Q fever/ C. burnetii | 2021 | Pakistan | Cross sectional | cattle, buffaloes | 827 Blood sample (cattle 419 and buffaloes 408) | The overall prevalence in dairy animals (cattle and buffaloes) was 6.1% (95% CI: 4.5-7.9). In cattle prevalence was higher (7.6%; 95% CI: 5.3-10.6) than in buffaloes (4.4%; 95% CI: 2.6-6.9) | Rashid, I.^109^ |  |
| Q fever/ C. burnetii | 2021 | Egypt | Cross sectional | sheep and goat | 91 blood sample (58 sheep and 33 goats) | We identified Coxiella burnetii in sheep and goats (1.7% and 3%) | Abdullah, Hham^110^ |  |
| Q fever/ C. burnetii | 2021 | Egypt | Cross sectional | cat | 40 serum samples | Out of 40 cats, 3 were positive for C. burnetii with an overall prevalence of 7.5%. | Abdel-Moein, K. A.^111^ |  |
| Q fever/ C. burnetii | 2021 | Somalia | Cross-sectional | Ticks | 237 | Prevalence 59.1% (140/237) of them were positive for Coxiella spp | Frangoulidis, D. ^112^ |  |

**REFERENCES**

1. SMS A. Prevalence of Coxiella burnetii infection among dogs and humans in upper Egypt. Assiut Veterinary Medical Journal. 2002; 47:205-15.

2. Scrimgeour EM, Al-Ismaily SI, Rolain JM, Al-Dhahry SH, El-Khatim HS, Raoult D. Q Fever in human and livestock populations in Oman. Annals of the New York Academy of Sciences. 2003; 990:221-5.

3. Kaabia N, Rolain JM, Khalifa M, Ben Jazia E, Bahri F, Raoult D, et al. Serologic study of rickettsioses among acute febrile patients in central Tunisia. Annals of the New York Academy of Sciences. 2006; 1078:176-9.

4. Faix DJ, Harrison DJ, Riddle MS, Vaughn AF, Yingst SL, Earhart K, et al. Outbreak of Q fever among US military in western Iraq, June-July 2005. Clinical infectious diseases : an official publication of the Infectious Diseases Society of America. 2008; 46:e65-8.

5. Khalili M, Sakhaee E. An update on a serologic survey of Q fever in domestic animals in Iran. American Journal of Tropical Medicine and Hygiene. 2009; 80:1031-2.

6. Sakhaee E, Khalili M. The first serologic study of Q fever in sheep in Iran. Tropical Animal Health and Production. 2010; 42:1561-4.

7. Azizzadeh M, Mohammadi GR, Haghparast AR, Heidarpour-Bami M. Seroepidemiology of Coxiella Burnetii in commercial dairy herds in northeast of Iran. Iranian Journal of Veterinary Science and Technology. 2011; 3:33-40.

8. Rahimi E. Coxiella burnetii in goat bulk milk samples in Iran. African Journal of Microbiology Research. 2010; 4:2324-6.

9. Khalili M, Shahabi-Nejad N, Golchin M. Q fever serology in febrile patients in southeast Iran. Transactions of The Royal Society of Tropical Medicine and Hygiene. 2010; 104:623-4.

10. Rahimi E, Ameri M, Karim G, Doosti A. Prevalence of Coxiella burnetii in bulk milk samples from dairy bovine, ovine, caprine, and camel herds in iran as determined by polymerase chain reaction. Foodborne Pathogens and Disease. 2011; 8:307-10.

11. Rahimi E, Doosti A, Ameri M, Kabiri E, Sharifian B. Detection of Coxiella burnetii by Nested PCR in bulk milk samples from dairy bovine, ovine, and caprine herds in Iran. Zoonoses and Public Health. 2010; 57:e38-e41.

12. Khalili M, Sakhaee E, Babaei H. Frequency of anti-Coxiella burnetii antibodies in cattle with reproductive disorders. Comparative Clinical Pathology. 2012; 21:917-9.

13. Abbasi S, Farzan R, Momtaz H. Molecular detection of Coxiella burnetii in goat bulk milk samples in some provinces of Iran. African Journal of Biotechnology. 2011; 10:18513-5.

14. Havas KA, Burkman K. A Comparison of the Serological Evidence of Coxiella burnetii Exposure Between Military Working Dogs and Feral Canines in Iraq. Military Medicine. 2011; 176:1101-3.

15. Anderson AD, Baker TR, Littrell AC, Mott RL, Niebuhr DW, Smoak BL. Seroepidemiologic Survey for Coxiella burnetii Among Hospitalized US Troops Deployed to Iraq*. Zoonoses and Public Health. 2011; 58:276-83.

16. Bailey MS, Trinick TR, Dunbar JA, Hatch R, Osborne JC, Brooks TJ, et al. Undifferentiated Febrile Illnesses Amongst British Troops in Helmand, Afghanistan. Journal of the Royal Army Medical Corps. 2011; 157:150-5.

17. Doosti A, Arshi A, Sadeghi M. Investigation of Coxiella burnetii in Iranian camels. Comparative Clinical Pathology. 2014; 23:43-6.

18. Mostafavi E, Esmaeili S, Shahdordizadeh M, Mahmoudi H, Liriayii H, Amiri FB. Seroepidemiological feature of Q fever among sheep in Northern Iran. Retrovirology. 2012; 9:P40.

19. Nahed HG, Abdel-Moein A, editors. Seroprevalence of Coxiella burnetii antibodies among farm animals and human contacts in Egypt2012.

20. POURMAHDI BORUJENI M, GHARIBI D, GOURANEJAD S, ZAMIRI S. SEROPREVALENCE OF COXIELLOSIS IN AHVAZ SHEEP. SCIENTIFIC-RESEARCH IRANIAN VETERINARY JOURNAL. 2013; 9:-.

21. Esmaeili S, Mostafavi E, Shahdordizadeh M, Mahmoudi H. A seroepidemiological survey of Q fever among sheep in Mazandaran province, northern Iran. Annals of Agricultural and Environmental Medicine. 2013; 20:708-10.

22. Asadi J, Kafi M, Khalili M. Seroprevalence of Q fever in sheep and goat flocks with a history of abortion in Iran between 2011 and 2012. Veterinaria Italiana. 2013; 49:163-8.

23. Ghalyanchi Langeroudi A, Babkhani N, Zolfaghari MR, Majidzadeh Arbadili K, Morovvati A, Soleimani M. Detection of Coxeilla brunetii in bulk tank milk samples from dairy bovine farms using nested-PCR in Qom, Iran, 2011. Iranian Journal of Veterinary Medicine. 2013; 7:207-11.

24. Kargar M, Rashidi A, Doosti A, Ghorbani-Dalini S, Najafi A. Prevalence of Coxiella burnetii in bovine bulk milk samples in southern Iran. Comparative Clinical Pathology. 2013; 22:331-4.

25. Esmaeili S, Pourhossein B, Gouya MM, Amiri FB, Mostafavi E. Seroepidemiological survey of Q fever and brucellosis in kurdistan Province, western Iran. Vector-Borne and Zoonotic Diseases. 2014; 14:41-5.

26. Gwida M, El-Ashker M, El-Diasty M, Engelhardt C, Khan I, Neubauer H. Q fever in cattle in some Egyptian Governorates: A preliminary study. BMC Research Notes. 2014; 7.

27. Borji s, Jamshidi a, khanzadi s, Razmyar J. Detection of Coxiella burnetii and sequencing the IS1111 gene fragment in bulk tank milk of dairy herds. Iranian Journal of Veterinary Science and Technology. 2014; 6:21-8.

28. Mohammed OB, Jarelnabi AA, Aljumaah RS, Alshaikh MA, Bakhiet AO, Omer SA, et al. Coxiella burnetii, the causative agent of Q fever in Saudi Arabia: Molecular detection from camel and other domestic livestock. Asian Pacific Journal of Tropical Medicine. 2014; 7:715-9.

29. khanzadi s, Jamshidi A, Razmyar J, Borji S. Identification of Coxiella burnetii by touch-down PCR assay in unpasteurized milk and dairy products in North - East of Iran. Iranian Journal of Veterinary Medicine. 2014; 8:15-9.

30. Peyman Khademi, Mohammad Reza Mahzounieh, Azizollah Ebrahimi Kahrizsangi, Emad Shdravan. Genomic detection of Coxiella burnetii in goat milk samples in animal farms Khorramabad Township, Iran. Pajoohande. 2014; 19:162-8.

31. KEYVANI RAD N, AZIZZADEH M, TAGHAVI RAZAVIZADEH AR, MEHRZAD J, RASHTIBAF M. SEROEPIDEMIOLOGY OF COXIELLOSIS (Q FEVER) IN SHEEP AND GOAT POPULATIONS IN THE NORTHEAST OF IRAN. IRANIAN JOURNAL OF VETERINARY RESEARCH (IJVR). 2014; 15:-.

32. Newman ENC, Johnstone P, Bridge H, Wright D, Jameson L, Bosworth A, et al. Seroconversion for Infectious Pathogens among UK Military Personnel Deployed to Afghanistan, 2008-2011. Emerging Infectious Diseases. 2014; 20:2015-22.

33. Khademi P, Mahzounieh M, Koutamehr ME. GENOMIC DETECTION OF COXIELLA BURNETII (Q FEVER AGENT) IN CATTLES MILK SAMPLES IN ANIMAL FARMS BONAB TOWNSHIP. Iran. Iranian Journal of Public Health. 2014; 43:292-.

34. Metanat M, Sepehri Rad N, Alavi-Naini R, Shahreki S, Sharifi-Mood B, Akhavan A, et al. Acute Q fever among febrile patients in Zahedan, southeastern Iran. Turkish Journal of Medical Sciences. 2014; 44:99-103.

35. Naderipour Z, Golchin M, Khalili M. Design of an ELISA Kit for Detection Human Acute Q Fever. Iranian Journal of Medical Microbiology. 2014; 8:28-34.

36. Aflatoonian MR, Khalili M, Sami M, Abiri Z. The frequency of IgM anti-Coxiella burnetii (Q fever) antibodies among slaughterhouse workers in Kerman city, 2012. Journal of Kerman University of Medical Sciences. 2014; 21:368-75.

37. Khalili M, Mosavi M, Diali HG, Mirza HN. Serologic survey for Coxiella burnetii phase II antibodies among slaughterhouse workers in Kerman, southeast of Iran. Asian Pacific Journal of Tropical Biomedicine. 2014; 4:S209-S12.

38. Khalili M, Qorbani A, Sharifi H, Golchin M. Prevalence and risk factor of Q fever among veterinary students in Iran. Tropical Biomedicine. 2015; 32:704-9.

39. Edalati-Shokat H, Abbasi-Doulatshahi E, Hajian-Bidar H, Gharekhani J, Rezaei A-A. Q fever in domestic ruminants: A Seroepidemiological survey in Hamedan, Iran. Int J Curr Microbiol App Sci. 2015; 4:589-96.

40. Khalili M, Diali HG, Mirza HN, Mosavi SM. Detection of Coxiella burnetii by PCR in bulk tank milk samples from dairy caprine herds in southeast of Iran. Asian Pacific Journal of Tropical Disease. 2015; 5:119-22.

41. Ahmadizadeh C, Moosakhani F, Jamshidian M. Detection and Identification of Coxiella burnetii in Milk Cattles of Tehran Province. 2015:48-52.

42. Janati Pirouz H, Mohammadi G, Mehrzad J, Azizzadeh M, Nazem Shirazi MH. Seroepidemiology of Q fever in one-humped camel population in northeast Iran. Tropical Animal Health and Production. 2015; 47:1293-8.

43. Ezatkhah M, Alimolaei M, Khalili M, Sharifi H. Seroepidemiological study of Q fever in small ruminants from Southeast Iran. Journal of Infection and Public Health. 2015; 8:170-6.

44. Akbarian Z, Ziay G, Schauwers W, Noormal B, Saeed I, Qanee AH, et al. Brucellosis and Coxiella burnetii Infection in Householders and Their Animals in Secure Villages in Herat Province, Afghanistan: A Cross-Sectional Study. PLoS Neglected Tropical Diseases. 2015; 9.

45. khademi p, Jaydari A, Esmaeili Koutamehr M. Genomic detection of Coxiella burnetii in cattle milk samples by Nested-PCR method, Iran. Iranian Journal of Medical Microbiology. 2015; 9:69-72.

46. Horton KC, Wasfy M, Samaha H, Abdel-Rahman B, Safwat S, Abdel Fadeel M, et al. Serosurvey for zoonotic viral and bacterial pathogens among slaughtered livestock in Egypt. Vector-Borne and Zoonotic Diseases. 2014; 14:633-9.

47. Fard SR N, Ghashghaei O, Khalili M, Sharifi H. Tick diversity and detection of Coxiella burnetii in tick of small ruminants using nested Trans PCR in Southeast Iran. Tropical Biomedicine. 2016; 33:506-11.

48. Zahid MU, Hussain MH, Saqib M, Neubauer H, Abbas G, Khan I, et al. Seroprevalence of Q Fever (Coxiellosis) in Small Ruminants of Two Districts in Punjab, Pakistan. Vector-Borne and Zoonotic Diseases. 2016; 16:449-54.

49. Khayyat Khameneie M, Asadi J, Khalili M, Abiri Z. The first serological study of Coxiella burnetii among pregnant women in Iran. Iranian Journal of Public Health. 2016; 45:523-30.

50. Janati MH, Mohammadi G, Mejrzad J, Azizzadeh M. Investigation of Coxiella burnetii Infection in Camel Population of Northeast of Iran with qPCR. Applied Animal Science Research Journal. 2017; 6:9-14.

51. Esmaeili S, Naddaf SR, Pourhossein B, Hashemi Shahraki A, Bagheri Amiri F, Gouya MM, et al. Seroprevalence of Brucellosis, Leptospirosis, and Q Fever among Butchers and Slaughterhouse Workers in South-Eastern Iran. PLoS One. 2016; 11:e0144953.

52. Rezaei A, Gh D. Seroprevalence of Lyme disease and Q fever in referred dogs to Veterinary Hospital of Ahvaz. Iranian Veterinary Journal. 2016; 11:34-41.

53. Farris CM, Pho N, Myers TE, Richards AL. Seroconversions for Coxiella and Rickettsial Pathogens among US Marines Deployed to Afghanistan, 2001-2010. Emerging Infectious Diseases. 2016; 22:1491-3.

54. KARIMIAN A, MAHZOUNIEH M, EBRAHIMI KAHRIZSANG A. GENOMIC DETECTION OF COXIELLA BURNETII IN BULK TANK MILK SAMPLES BY NESTED-PCR METHOD IN SHAHREKORD, IRAN. RESEARCHER BULLETIN OF MEDICAL SCIENCES (PEJOUHANDEH). 2016; 21:-.

55. Lorestani S, Jaydari A, Maleki S, Khademi P. Genomic detection of Coxiella burnetii in sheep milk samples by Nested-PCR method in Khorramabad, Iran. Journal of food science and technology(Iran). 2015; 13:165-71.

56. Abushahba MFN, Abdelbaset AE, Rawy MS, Ahmed SO. Cross-sectional study for determining the prevalence of Q fever in small ruminants and humans at El Minya Governorate, Egypt. BMC Research Notes. 2017; 10.

57. Ghashghaei O, Nourollahi-Fard SR, Khalili M, Sharifi H. A survey of ixodid ticks feeding on cattle and molecular detection of Coxiella burnetii from ticks in Southeast Iran. Turkish Journal of Veterinary and Animal Sciences. 2017; 41:46-50.

58. Abdel-Moein KA, Hamza DA. The burden of Coxiella burnetii among aborted dairy animals in Egypt and its public health implications. Acta Tropica. 2017; 166:92-5.

59. Khalafalla AI, Al Eknah MM, Abdelaziz M, Ghoneim IM. A study on some reproductive disorders in dromedary camel herds in Saudi Arabia with special references to uterine infections and abortion. Tropical Animal Health and Production. 2017; 49:967-74.

60. Obaidat MM, Kersh GJ. Prevalence and Risk Factors of Coxiella burnetii Antibodies in Bulk Milk from Cattle, Sheep, and Goats in Jordan. J Food Prot. 2017; 80:561-6.

61. Hosseinzadeh S, Oryan A, Limaki SK, Moaddeli A, Poormontaseri M, Taghadosi V. Molecular characterization of Coxiella burnetii in the slaughtered animals of Southern Iran. Asian Pacific Journal of Tropical Disease. 2017; 7:753-6.

62. Ghasemian R, Mostafavi E, Esmaeili S, Najafi N, Arabsheybani S. A Survey of Acute Q Fever among Patients with Brucellosis-Like and Atypical Pneumonia Symptoms Who Are Referred to Qaemshahr Razi Hospital in Northern Iran (2014–2015). Global Journal of Health Science. 2016; 9:225-32.

63. Aflatoonian MR, Khalili M, Rahanjam M, Aflatoonian B. Q fever seroepidemiology and associated risk factors in veterinarians and vet staff in Southern Khorasan, Iran, 2014. Iranian Journal of Epidemiology. 2016; 11:38-45.

64. Nokhodian Z, Ataei B, Moradi A, Yaran M, Hoseini SG, Feizi A, et al. Seroprevalence and risk factors of Coxiella burnetii infection among high-risk population in center of Iran, a neglected health problem. Acta Tropica. 2017; 169:107-11.

65. Esmailnejad A, Hasiri MA. Serological evidence of Coxiella burnetii infection among companion dogs in Fars province, South Iran. Bulgarian Journal of Veterinary Medicine. 2017; 20:377-84.

66. Kayedi MH, Mokhayeri H, Birjandi M, Chegeni-Sharafi A, Esmaeili S, Mostafavi E. Seroepidemiological study of Q fever in Lorestan province, western Iran, 2014. Iranian Journal of Microbiology. 2017; 9:213-8.

67. Esmaeili S, Golzar F, Ayubi E, Naghili B, Mostafavi E. Acute Q fever in febrile patients in northwestern of Iran. PLoS Neglected Tropical Diseases. 2017; 11.

68. Nokhodian Z, Ataei B, khalili M, Feizi A, Moradi A, Yaran M, et al. Detection of Coxiella burnetii and risk factors for infection in ruminants in a central county of Iran. Veterinary Microbiology. 2018; 222:7-10.

69. Nokhodian Z, Feizi A, Khalili M, Ataei B, Moradi A. Molecular evidence and risk factors of Coxiella burnetii among seropositive high-risk individuals in the center of Iran. Comparative Immunology, Microbiology and Infectious Diseases. 2018; 61:34-7.

70. Khalili M, Rezaei M, Akhtardanesh B, Abiri Z, Shahheidaripour S. Detection of Coxiella burnetii (Gammaproteobacteria: Coxiellaceae) in ticks collected from infested dogs in Kerman, Southeast of Iran. Persian Journal of Acarology. 2018; 7:93-100.

71. Abdullah HHAM, El-Shanawany EE, Abdel-Shafy S, Abou-Zeina HAA, Abdel-Rahman EH. Molecular and immunological characterization of Hyalomma dromedarii and Hyalomma excavatum (Acari: Ixodidae) vectors of Q fever in camels. Veterinary World. 2018; 11:1109-19.

72. Klemmer J, Njeru J, Emam A, El-Sayed A, Moawad AA, Henning K, et al. Q fever in Egypt: Epidemiological survey of Coxiella burnetii specific antibodies in cattle, buffaloes, sheep, goats and camels. PLoS ONE. 2018; 13.

73. Jarelnabi AA, Alshaikh MA, Bakhiet AO, Omer SA, Aljumaah RS, Harkiss GD, et al. Seroprevalence of q fever in farm animals in saudi arabia. Biomedical Research (India). 2018; 29:895-900.

74. Alhetheel AF, Binkhamis K, Somily A, Barry M, Shakoor Z. Screening for Q fever. A tertiary care hospital-based experience in central Saudi Arabia. Saudi Med J. 2018; 39:1195-9.

75. Barkallah M, Gharbi Y, Hmani M, Mallek Z, Gautier M, Gdoura R, et al. Serological and molecular evidence of coxiellosis and risk factors in sheep flocks in central-eastern Tunisia. Comparative Immunology, Microbiology and Infectious Diseases. 2018; 57:15-21.

76. Messous S, Grissa MH, Beltaief K, Boukef R, Nouira S, Mastouri M. Bacteriology of acute exacerbations of chronic obstructive pulmonary disease in Tunisia. Revue des Maladies Respiratoires. 2018; 35:36-47.

77. Selmi R, Mamlouk A, Ben Yahia H, Abdelaali H, Ben Said M, Sellami K, et al. Coxiella burnetii in Tunisian dromedary camels (Camelus dromedarius): Seroprevalence, associated risk factors and seasonal dynamics. Acta Trop. 2018; 188:234-9.

78. Esmaeili S, Mohabati Mobarez A, Khalili M, Mostafavi E, Moradnejad P. Genetic evidence of Coxiella burnetii infection in acute febrile illnesses in Iran. PLoS Negl Trop Dis. 2019; 13:e0007181.

79. Ghasemian R, Mostafavi E, Esmaeili S, Arabsheybani S, Davoodi L. Epidemiologic investigation of acute q fever in North of Iran. Journal of Mazandaran University of Medical Sciences. 2019; 29:100-6.

80. Khademi P, Ownagh A, Mardani K, Khalili M. Prevalence of Coxiella burnetii in milk collected from buffalo (water buffalo) and cattle dairy farms in Northwest of Iran. Comparative Immunology, Microbiology and Infectious Diseases. 2019; 67.

81. Abdullah HHAM, Hussein HA, Abd El-Razik KA, Barakat AMA, Soliman YA. Q fever: A neglected disease of camels in Giza and Cairo Provinces, Egypt. Veterinary World. 2019; 12:1945-50.

82. Byomi A, Zidan S, Elkamshishi M, Sakr M, Elsify A, Eissa N, et al. Some associated risk factors with Coxiella burnetii in sheep, humans and ticks in Menoufiya governorate, Egypt. Bioscience Research. 2019; 16:121-38.

83. Selmi R, Ben Said M, Mamlouk A, Ben Yahia H, Messadi L. Molecular detection and genetic characterization of the potentially pathogenic Coxiella burnetii and the endosymbiotic Candidatus Midichloria mitochondrii in ticks infesting camels (Camelus dromedarius) from Tunisia. Microbial Pathogenesis. 2019; 136.

84. Esmaeili S, Bagheri Amiri F, Mokhayeri H, Kayedi MH, Maurin M, Rohani M, et al. Seroepidemiological study of Q fever, brucellosis and tularemia in butchers and slaughterhouses workers in Lorestan, western of Iran. Comparative Immunology, Microbiology and Infectious Diseases. 2019; 66.

85. Moradnejad P, Esmaeili S, Maleki M, Sadeghpour A, Kamali M, Rohani M, et al. Q Fever Endocarditis in Iran. Scientific Reports. 2019; 9.

86. Obaidat MM, Malania L, Imnadze P, Roess AA, Bani Salman AE, Arner RJ. Seroprevalence and Risk Factors for Coxiella burnetii in Jordan. The American journal of tropical medicine and hygiene. 2019; 101:40-4.

87. Mostafavi E, Molaeipoor L, Esmaeili S, Ghasemi A, Kamalizad M, Behzadi MY, et al. Seroprevalence of Q fever among high-risk occupations in the Ilam province, the west of Iran. PLoS ONE. 2019; 14.

88. Esmaeili S, Mobarez AM, Khalili M, Mostafavi E. High prevalence and risk factors of Coxiella burnetii in milk of dairy animals with a history of abortion in Iran. Comparative Immunology Microbiology and Infectious Diseases. 2019; 63:127-30.

89. Ullah Q, El-Adawy H, Jamil T, Jamil H, Qureshi ZI, Saqib M, et al. Serological and molecular investigation of Coxiella burnetii in small ruminants and ticks in Punjab, Pakistan. International Journal of Environmental Research and Public Health. 2019; 16.

90. Barigye R, Hassan NA, Alqubaisi DMN, Abdalla-Alfaki IM. Serological evidence of coxiella burnetii, leptospira interrogans hardjo, neospora caninum and bovine pestivirus infections in a dairy cattle herd from the United Arab Emirates. Veterinaria Italiana. 2020; 56:163-8.

91. Ahmadi E. Potential public health risk due to consumption of contaminated bovine milk with aflatoxin M1 and Coxiella burnetii in the West of Iran. International Journal of Dairy Technology. 2020; 73:479-85.

92. Khademi P, Ownagh A, Ataei B, Kazemnia A, Enferadi A, Khalili M, et al. Prevalence of C. burnetii DNA in sheep and goats milk in the northwest of Iran. International Journal of Food Microbiology. 2020; 331.

93. Khademi P, Ownagh A, Ataei B, Kazemnia A, Eydi J, Khalili M, et al. Molecular detection of Coxiella burnetii in horse sera in Iran. Comparative Immunology Microbiology and Infectious Diseases. 2020; 72.

94. Mousapour M, Oveisi A, Key YA, Mikaeili E, Rahimi F, Shademan B, et al. First Serological & Molecular Study of Coxiella burnetii in Stray, Domestic Cats, and Their Owners in Iran. Top Companion Anim Med. 2020; 41:100471.

95. Selim A, Ali AF. Seroprevalence and risk factors for C. burentii infection in camels in Egypt. Comparative Immunology, Microbiology and Infectious Diseases. 2020; 68.

96. Lafi SQ, Talafha AQ, Abu-Dalbouh MA, Hailat RS, Khalifeh MS. Seroprevalence and associated risk factors of Coxiella burnetii (Q fever) in goats and sheep in northern Jordan. Tropical Animal Health and Production. 2020; 52:1553-9.

97. Hussien MO, ElFahal AM, Enan KA, Taha KM, Mohammed MS, Salih DA, et al. Seroprevalence of Q fever in Goats in the Sudan. Veterinary World. 2012; 5:394-7.

98. Aljafar A, Salem M, Housawi F, Zaghawa A, Hegazy Y. Seroprevalence and risk factors of Q-fever (C. burnetii infection) among ruminants reared in the eastern region of the Kingdom of Saudi Arabia. Tropical Animal Health and Production. 2020; 52:2631-8.

99. Gharban HAJ, Yousif AA. SEROLOGICAL, CLINICAL AND HEMATOLOGICAL PREVALENCE OF COXIELLA BURNETII IN ADULT COWS, IRAQ. Biochemical and Cellular Archives. 2020; 20:67-74.

100. Dabaja MF, Greco G, Blanda V, Tempesta M, Bayan A, Torina A, et al. Multispacer sequence typing of coxiella burnetii from milk and hard tick samples from ruminant farms in Lebanon. Veterinaria Italiana. 2020; 56:289-96.

101. Abbass H, Selim SAK, Sobhy MM, El-Mokhtar MA, Elhariri M, Abd-Elhafeez HH. High prevalence of Coxiella burnetii infection in humans and livestock in Assiut, Egypt: A serological and molecular survey. Veterinary World. 2020; 13:2578-86.

102. Mobarez AM, Mostafavi E, Khalili M, Esmaeili S. Identification of Coxiella burnetii in Raw Milk of Livestock Animal in Iran. International Journal of Microbiology. 2021; 2021.

103. Jaferi M, Mozaffari A, Jajarmi M, Imani M, Khalili M. Serologic and molecular survey of horses to Coxiella burnetii in East of Iran a highly endemic area. Comparative Immunology, Microbiology and Infectious Diseases. 2021; 76.

104. Sabzevari S, Shoraka H, Seyyedin M. Seroepidemiological survey of brucellosis and q fever among high-risk occupations in northeast of iran for first time. Iranian Journal of Microbiology. 2021; 13:325-36.

105. fakour S, Jamali R, Ahmadi E. Seroepidemiological study on Coxiella burnetii and associated risk factors in ruminants at Kurdistan Province, west of Iran. Comparative Immunology, Microbiology and Infectious Diseases. 2021; 78.

106. El Tigani-Asil EA, Blanda V, Abdelwahab GE, Al Hammadi ZM, Habeeba S, Khalafalla AI, et al. Molecular Investigation on Tick-Borne Hemoparasites and Coxiella burnetii in Dromedary Camels (Camelus dromedarius) in Al Dhafra Region of Abu Dhabi, UAE. Animals. 2021; 11.

107. Barigye R, Hassan NAD, Abdalla Alfaki IM, Barongo MB, Mohamed MEH, Mohteshamuddin K. Seroprevalence of Coxiella burnetii in a dairy cattle herd from the Al Ain region, United Arab Emirates. Tropical Animal Health and Production. 2021; 53.

108. Iqbal MZ, Durrani AZ, Khan JA, Ahmad N, Usman M, Jabbar A, et al. Molecular epidemiology of Coxiella Brunetii in small ruminants in Punjab, Pakistan: a novel reporting analytical cross sectional study. Tropical Animal Health and Production. 2021; 53.

109. Rashid I, Saqib M, Ahmad T, Sajid MS. Sero-Prevalence and Associated Risk Factors of Q Fever in Cattle and Buffaloes Managed at Institutional Dairy Farms. Pakistan Veterinary Journal. 2019; 39:221-5.

110. Abdullah H, Amanzougaghene N, Dahmana H, Louni M, Raoult D, Mediannikov O. Multiple vector-borne pathogens of domestic animals in Egypt. Plos Neglected Tropical Diseases. 2021; 15.

111. Abdel-Moein KA, Zaher HM. Parturient Cat As a Potential Reservoir for Coxiella burnetii: A Hidden Threat to Pet Owners. Vector-Borne and Zoonotic Diseases. 2021; 21:264-8.

112. Frangoulidis D, Kahlhofer C, Said AS, Osman AY, Chitimia-Dobler L, Shuaib YA. High prevalence and new genotype of coxiella burnetii in ticks infesting camels in somalia. Pathogens. 2021; 10.
